# The impact of tokenizer selection in genomic language models

**DOI:** 10.1093/bioinformatics/btaf456

**Published:** 2025-08-18

**Authors:** LeAnn M Lindsey, Nicole L Pershing, Anisa Habib, Keith Dufault-Thompson, W Zac Stephens, Anne J Blaschke, Xiaofang Jiang, Hari Sundar

**Affiliations:** Kahlert School of Computing, University of Utah, Salt Lake City, Utah 84112, United States; National Library of Medicine, National Institutes of Health, Bethesda, Maryland 20892, United States; Department of Pediatrics, School of Medicine, University of Utah, Salt Lake City, Utah 84112, United States; Kahlert School of Computing, University of Utah, Salt Lake City, Utah 84112, United States; National Library of Medicine, National Institutes of Health, Bethesda, Maryland 20892, United States; Department of Pathology, School of Medicine, University of Utah, Salt Lake City, Utah 84112, United States; Department of Pediatrics, School of Medicine, University of Utah, Salt Lake City, Utah 84112, United States; National Library of Medicine, National Institutes of Health, Bethesda, Maryland 20892, United States; Department of Computer Science, Tufts University, Medford, MA 02115, United States

## Abstract

**Motivation:**

Genomic language models have recently emerged as a new method to decode, interpret, and generate genetic sequences. Existing genomic language models have utilized various tokenization methods, including character tokenization, overlapping and nonoverlapping k-mer tokenization, and byte-pair encoding, a method widely used in natural language models. Genomic sequences differ from natural language because of their low character variability, complex and overlapping features, and inconsistent directionality. These features make subword tokenization in genomic language models significantly different from both traditional language models and protein language models.

**Results:**

This study explores the impact of tokenization in genomic language models by evaluating their downstream performance on 44 classification fine-tuning tasks. We also perform a direct comparison of byte pair encoding and character tokenization in Mamba, a state-space model. Our results indicate that character tokenization outperforms subword tokenization methods on tasks that rely on nucleotide-level resolution, such as splice site prediction and promoter detection. While byte-pair tokenization had stronger performance on the SARS-CoV-2 variant classification task, we observed limited statistically significant differences between tokenization methods on the remaining downstream tasks.

**Availability and implementation:**

Detailed results of all benchmarking experiments are available in https://github.com/leannmlindsey/DNAtokenization. Training datasets and pretrained models are available at https://huggingface.co/datasets/leannmlindsey. Datasets and processing scripts are available at doi: 10.5281/zenodo.16287401 and doi: 10.5281/zenodo.16287130.

## 1 Introduction

Tokenization is a fundamental step in the language model preprocessing pipeline and is used to parse an input sequence into segments called *tokens* that represent either words, subwords, or characters. These tokens are assigned numeric values and used as inputs to a neural network in order to learn context-specific embeddings. While subword tokenization methods have become a de facto standard in large language models (Ngo Ho and Yvon 2021, Berglund and van der Merwe 2023), various genomic language models (gLMs) have adopted different tokenization approaches, with no consensus emerging in the field. As gLMs continue to advance, it will be essential to understand the performance impact of tokenization on downstream biological tasks.

**Figure 1.**
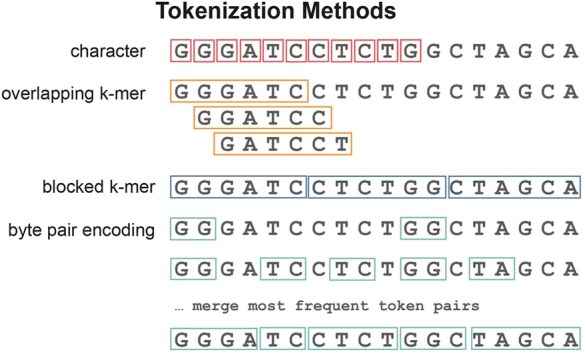
Sample text processed using different tokenization strategies: character, overlapping k-mer, non-overlapping k-mer, and byte-pair encoding.

Current gLMs have been trained using three main tokenization methods: *character-based tokenization*, in which the sequences are tokenized by individual nucleotides, *k-mer tokenization*, where the input is tokenized into either overlapping or nonoverlapping substrings of length *k*, and subword tokenization using *byte-pair encoding* (Fig. 1).

Byte pair encoding (BPE) was originally developed as a method of compression and was later widely adopted by the natural language processing (NLP) community as a method of tokenization for language models. BPE iteratively calculates the frequency of adjacent characters and merges the most frequent pairs, using these frequencies to construct a vocabulary of the most frequently seen subwords in a training corpus. This creates a compact representation that allows models to capture semantic relationships, reduce vocabulary size, and handle rare and out-of-vocabulary words. BPE also provides significant text compression benefits, with the DNABERT-2 tokenizer achieving 4–5× compression ratios (Zhou *et al.* 2024). The nonoverlapping k-mer tokenization used by the Nucleotide Transformer model, which we label *blocked k-mer*, also provides this same compression advantage. This efficient representation expands the effective context window capacity of the model, reducing training time and, subsequently, the cost to train the model.

Though subword tokenization provides many benefits, it also introduces several challenges that have been shown to adversely affect model performance in natural language models. Inconsistent tokenization, where the same word can be tokenized differently based on its position in the text, can lead to performance degradation, model hallucinations (Bostrom and Durrett 2020, Sun *et al.* 2023), and a loss of semantic relationships between subwords (Batsuren *et al.* 2024). Lack of character-level transparency when using subword tokenization can also cause difficulty with tasks that require character-level reasoning such as precise spelling, letter counting, and arithmetic (Bostrom and Durrett 2020, Chai *et al.* 2024).

Despite subword tokenization being broadly used in natural language processing, the ideal tokenization method is still being actively explored, with substantial research dedicated to understanding the impact of various tokenization choices (Rust *et al.* 2021, Sun *et al.* 2023, Rajaraman *et al.* 2024, Schmidt *et al.* 2024, Singh and Strouse 2024). Similar studies are needed in the genomic language domain, where the content and complexity differ significantly from natural language. Compared to natural language and protein sequences, nucleotide sequences have low character variability, contain overlapping and nested regulatory features, and have no obvious “word” demarcations. These differences suggest that the commonly used approaches for tokenization in natural language need to be evaluated in the significantly different context of nucleotide sequences.

The most significant work studying tokenization in biological language models to date is Dotan *et al.* (2024), which tested five different tokenizers of various sizes: BPE (Sennrich *et al.* 2016), Unigram (Kudo 2018), WordPiece (Schuster and Nakajima 2012), characters, and pairs, against eight different biological datasets. They concluded that the choice of tokenizer has a significant impact on the downstream accuracy of the model, observing a change in accuracy as much as 5% and a change in MCC score as large as 0.10 depending on the tokenizer/task combination. Their experiments primarily focused on models trained on amino acid sequences, with only one experiment using nucleotide sequences as input, leaving open questions about the downstream impact of tokenization on nucleotide sequences.

Various researchers have discussed the impact of tokenization when introducing new gLMs, providing anecdotal evidence about the benefits and drawbacks of different approaches. The authors of DNABERT-2 compared BPE and k-mer tokenization in an ablation study, concluding that BPE performed better on average, although their experiments did indicate that k-mer tokenization had better performance in some tasks, including promoter detection (Zhou *et al.* 2024). The authors of HyenaDNA (Nguyen *et al.* 2023) compared BPE with k-mer tokenization and concluded that using BPE tokenization with their model degraded their results. Schiff *et al.* (2024) observed that with k-mer tokenization, small changes in the input sequence can result in dramatic changes in tokenization, but published no experiments linking these tokenization changes to downstream performance. These studies demonstrate that tokenization choices can have significant downstream impact on model performance, highlighting the need for a systematic analysis of tokenization in the genomic language domain.

In this study, we investigate the following questions:

- Is subword tokenization in gLMs simply a form of compression, or does tokenization assist the model in capturing meaningful contextual relationships between tokens?
- How does the choice of tokenizer impact model performance on downstream tasks?

In order to investigate these questions, we compared three attention-based gLMs, three state space gLMs, and two baseline models on 44 downstream classification tasks compiled from three published genomic benchmarks, the Genomic Benchmark (GB) (Grešová *et al.* 2023), the Nucleotide Transformer Tasks (revised) (NTTv2) (Dalla-Torre *et al.* 2024), and the Genome Understanding Evaluation (GUE) (Zhou *et al.* 2024). We also trained a 4-layer Mamba state space model using both byte-pair encoding and character tokenization to gauge the direct impact of tokenization choice on model performance.

The results of our study indicate that the choice of tokenization method can significantly impact model performance, particularly on tasks that require the detection of specific nucleotide motifs. This work highlights the need for tailored machine learning approaches for biological applications that optimize pairing between the best-suited tokenization methods and the biological feature(s) being investigated.

## 2 Materials and methods

### 2.1 Genomic language models

We tested two baseline models, a simple three-layer CNN (Grešová *et al.* 2023) trained using one-hot encoding and GPT-Neo-125m from EleutherAI. Although the GPT-Neo model was not trained on DNA, it performed significantly better than random so we include it as a second baseline comparison. GPT-Neo uses BPE tokenization, and no changes were made to the model or tokenizer to adapt them to DNA for these experiments.

We compared the transformer-based models, Nucleotide Transformer version 2 (nucleotide-transformer-v2-500m-multi-species) (Dalla-Torre *et al.* 2024), DNABERT (Ji *et al.* 2021), and DNABERT-2 (Zhou *et al.* 2024), against three of the state space gLMs, HyenaDNA (Nguyen *et al.* 2023), Mamba (Gu and Dao 2024), and Caduceus (Schiff *et al.* 2024). To directly compare the effect of a tokenizer on a state space model, we pretrained a 4-layer Mamba model using an input sequence length of 4096, dimension 256, using both character and BPE tokenization. More details on this model and our hyperparameter tuning during pretraining are available in the Supplementary Material, available as supplementary data at *Bioinformatics* online. All of the state space models were trained using the same human reference genome (Hg38) dataset used to train the Caduceus model (Schiff *et al.* 2024) and used a context length of 4000 nucleotides. Details on all models can be seen in Table 1.

**Table 1.** Details of all models used in the benchmarking experiments.

Model	Architecture	Parameters	Tokenization	Max context window (nt)	Pretraining data	Pretraining time	Pretraining hardware
CNN	CNN	464 K	one-hot encoding	N/A	N/A	N/A	N/A
GPT-Neo	Attention	125 M	BPE	2048	The Pile	unknown	Google TPUs
Nucleotide Transformer	Attention	500 M	block k-mer	512	3202 Genetically Diverse Human Genomes	14 days	16x 8 Nvidia A100
DNABERT	Attention	117 M	overlap k-mer	512	Hg38 Human Reference Genome	25 days	8 Nvidia 2080 Ti
DNABERT-2	Attention	117 M	BPE	∼2500	Human Genome and 135 Other Species	14 days	8 Nvidia 2080 Ti
HyenaDNA	State Space	13.1 M	char	1M	Hg38 Human Reference Genome	80 min	1 Nvidia A100
Mamba	State Space	1.8 M	char	131K	Hg38 Human Reference Genome	4–12 h	4 Nvidia A100
Caduceus	State Space Equivariant	3.9 M	char	131K	Hg38 Human Reference Genome	4–12 h	4 Nvidia A100

A simple 3-layer CNN was used as a baseline model. The GPT-Neo pretrained model was used as a second baseline. The Time and Hardware columns specify the hardware used and total time needed for pretraining.

### 2.2 Benchmarks

We benchmarked all models against three published genomic fine-tuning benchmarks: the recently revised Nucleotide Transformer Tasks (Dalla-Torre *et al.* 2024), the Genomic Benchmark (Grešová *et al.* 2023), and the Genome Understanding Evaluation (Zhou *et al.* 2024). All fine-tuning tasks were replicated a minimum of 10 times. A hyperparameter search for the best batch size and learning rate was performed using three initial seed values for the state space models since they are sensitive to these parameters. For the attention-based models, we used the learning rate and batch sizes reported in the fine-tuning experiments in Zhou *et al.* (2024). Details are available in the Supplementary Material, available as supplementary data at *Bioinformatics* online.

### 2.3 Metrics

The mean accuracy and Mathews Correlation Coefficient (MCC) were reported on each fine-tuning task.


(1) $$\mathrm{MCC}=\frac{TP\times TN-FP\times FN}{\sqrt{\left(TP+FP)(TP+FN)(TN+FP)(TN+FN\right)}}$$

The MCC score ranges from −1 to 1 with 1 representing perfect prediction and 0 representing random performance. In the case of tasks that have more than two labels, the macro average was reported, which gives equal weight to each class regardless of the number of members of each class.

### 2.4 Statistical testing

To evaluate the significance of the performance differences between the Mamba-char and Mamba-bpe models, we performed paired *t*-tests, pairing replicates with identical random seeds. We performed tests at both the task and task category aggregation levels. To address multiple testing concerns arising from categorical comparisons, we applied Bonferroni correction (Dunn 1959) at each level to control the family-wise error rate. For individual task comparisons, we used α=0.05/number of tasks and for category-level comparisons, we used α=0.05/number of categories. Bonferroni correction is a conservative approach that reduces the significance threshold to prevent inflation of statistical significance that can occur when multiple tests are performed on the same dataset.

### 2.5 Tokenizers

Our experiments on tokenization were implemented using the transformers library from *HuggingFace* (Wolf *et al.* 2020). We limited this study to token vocabularies of size 4096 because of the experiments by Zhou *et al.* (2024) that recommended tokenized vocabularies of this size for gLMs.

### 2.6 Genomic tasks

The genomic tasks in the benchmarking datasets can be grouped into nine categories: regulatory elements, promoter detection, enhancer detection, transcription factor binding site prediction, epigenetic marks prediction, splice site detection, coding region detection, taxonomic classification, and virus variant detection.

The regulatory elements category includes Ensembl reference datasets annotating promoters, enhancers, and open chromatin regions (Zerbino *et al.* 2015, Howe *et al.* 2021). Promoters are regions typically located upstream of gene transcription start sites and serve as platforms for RNA polymerase and transcription factors to initiate gene expression; these can be further subclassified by specific genomic sequence features. Enhancers are regulatory elements that can be located upstream, downstream, or within the introns of their target genes, often acting over long distances. Open chromatin regions are genomic regions accessible for protein binding that lack evidence to classify them as promoters or enhancers.

Transcription factor binding sites contain motifs ranging from 6 to 20 nt in length, where transcription factors bind to regulate gene expression. They are usually located within promoter, enhancer, or other regulatory regions. These motifs typically display sequence flexibility, allowing inexact matches to the consensus motif, with the binding affinity correlating with the similarity to the optimal binding motif (Spitz and Furlong 2012).

Open chromatin regions are genomic regions that are accessible to DNA-binding proteins due to relaxed nucleosome packing. Chromatin accessibility and nucleosome packing are regulated by multiple epigenetic mechanisms, including post-translational modification of histone tails (such as acetylation and methylation), which alter nucleosome structure and chromatin accessibility. The epigenetic marks prediction task focuses on identifying regions associated with specific histone modifications that regulate expression.

Splice sites are specific sequences at exon–intron boundaries that direct the splicing machinery and typically include canonical GT dinucleotides at the 5′ donor sites and AG dinucleotides at the 3′ acceptor sites.

Tasks that did not align with other genomic feature categories—including coding versus intergenic, worm versus human, and SARS-CoV-2 variant classification—were maintained as standalone categories.

## 3 Results

### 3.1 Tokenization differentially impacts performance of gLMs on specific benchmark tasks

**Figure 2.**
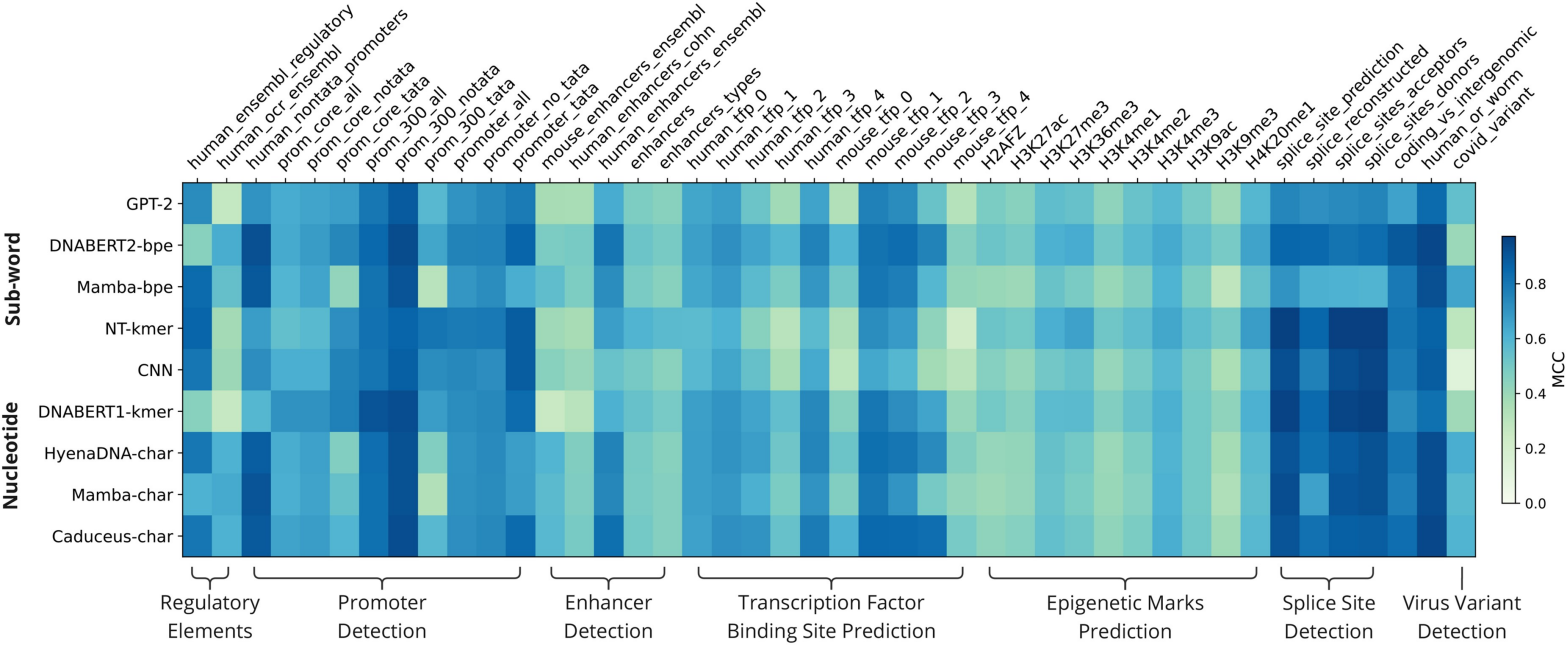
Model performance as measured by the Matthews Correlation Coefficient on all benchmark tasks, grouped by task category. MCC score is visualized as a gradient, with darker shading indicating better performance. The rows are clustered by the type of tokenization method, with subword tokenization near the top followed by the nucleotide-level tokenization methods.

The best performing model in each task category varied significantly, with the Nucleotide Transformer having the highest performance on the splice sites and epigenetic marks detection tasks (Fig. 2, Table 2). The original DNABERT model had the highest MCC score on the promoter detection task, Caduceus had the highest performance on regulatory elements, the enhancer detection and transcription factor binding site prediction tasks. The Mamba-bpe model had the best performance on the SARS-CoV-2 virus variant detection task. Caduceus had the best performance overall, followed closely by DNABERT2.

**Table 2.** Overview of MCC scores across models summarized by task category and benchmark.

Category	CNN	GPT-Neo	DNABERT-2 (bpe)	Mamba (bpe)	NT (blocked k-mer)	DNABERT (k-mer)	HyenaDNA (char)	Mamba (char)	Caduceus (char)
Model size (parameters)	464K	125M	117M	3.9M	500M	117M	13.1M	1.8M	3.9M
Regulatory	0.638	0.568	0.664	0.760	0.634	0.431	0.760	0.716	**0.778**
Promoters	0.749	0.716	0.762	0.639	0.754	**0.777**	0.675	0.667	0.728
Enhancers	0.467	0.457	0.559	0.539	0.520	0.441	0.552	0.548	**0.573**
Transcription factors	0.496	0.557	0.685	0.616	0.490	0.635	0.667	0.605	**0.703**
Epigenetic marks	0.480	0.504	0.568	0.460	**0.576**	0.526	0.481	0.470	0.501
Splice sites	0.893	0.738	0.835	0.631	**0.941**	0.931	0.884	0.854	0.870
Virus variant detection	0.128	0.549	0.401	**0.652**	0.498	0.389	0.620	0.576	0.630
GUE	0.569	0.614	0.699	0.618	0.587	0.682	0.671	0.621	**0.707**
Genomic benchmark	0.622	0.570	0.702	0.719	0.625	0.502	0.729	0.705	**0.750**
Nucleotide transformer tasks	0.606	0.579	0.643	0.527	**0.684**	0.638	0.588	0.585	0.608
Overall	0.594	0.583	0.673	0.599	0.634	0.617	0.643	0.621	**0.674**
	*Baseline*		*Subword tokenization*			*Nucleotide level tokenization*			

The highest performing model in each row is highlighted in bold and underlined. The benchmarks included are: Genomic Benchmark (GB) (Grešová *et al.* 2023), Nucleotide Transformer Tasks (NTT) (Dalla-Torre *et al.* 2024), GUE (Genome Understanding Evaluation) (Zhou *et al.* 2024).

**Figure 3.**
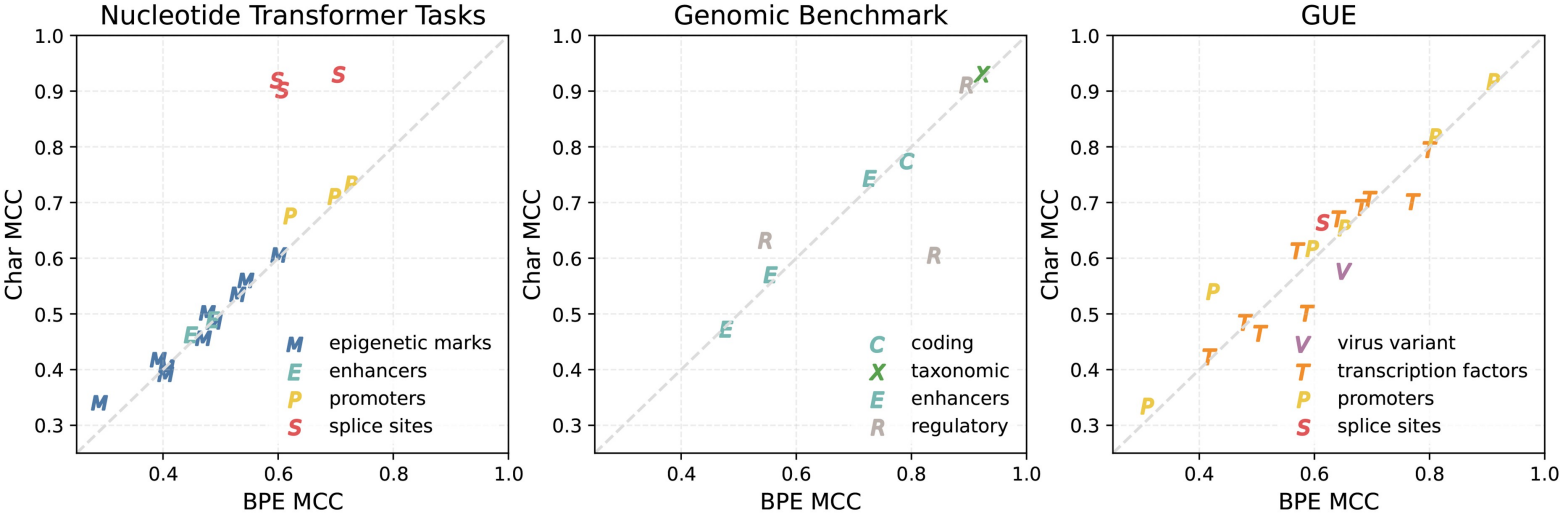
Comparison of tokenization methods in a 4-layer Mamba-DNA model on all three genomic benchmarks. Markers above the diagonal line indicate that the Mamba model with character tokenization has higher MCC score than BPE tokenization on the same dataset. Markers are colored by task category.

In the direct comparison experiment using the Mamba model, the different tokenization approaches showed significantly different performance on a subset of the biological tasks (Fig. 3, Tables 3 and 4). In the promoters category, the mean difference was 0.0289 (t=6.09, p<0.0001) with character tokenization having better performance on this task. In the splice site detection category, character tokenization shows significantly better performance with a mean MCC difference of 0.2235 (t=12.44, p<0.0001). In the virus variant detection task, byte-pair encoding had better performance with a mean difference of 0.0726 (t=7.17, p<0.0002). We observe a slight but statistically significant difference in favor of character tokenization on the epigenetic marks and taxonomic task categories. In the coding, enhancers and transcription factor categories, we do not observe a statistically significant difference between tokenizers.

**Table 3.** Task-level paired comparison of character-level versus byte pair encoding tokenization on MCC scores in a 4-layer Mamba-DNA model (matched by seed).

Task	n	t-stat	*P*-value	Mean diff (char—bpe)
**Coding**				
Coding vs intergenomic	10	−0.664	0.523	−0.029
**Enhancers**				
Dummy mouse enhancers	10	0.895	0.394	0.016
Enhancers	10	0.586	0.572	0.002
Enhancers types	10	3.338	0.009**	0.017
Human enhancers cohn	10	−1.902	0.090	−0.006
Human enhancers ensembl	10	0.229	0.824	0.004
**Epigenetic marks**				
H2AFZ	10	−2.231	0.053	−0.014
H3K27ac	10	5.478	0.000^***†^	0.033
H3K27me3	10	−0.102	0.921	−0.000
H3K36me3	10	−0.743	0.477	−0.004
H3K4me1	10	0.726	0.486	0.002
H3K4me2	10	−2.536	0.032*	−0.019
H3K4me3	10	0.950	0.367	0.006
H3K9ac	10	4.906	0.001^***†^	0.035
H3K9me3	10	4.202	0.002**	0.036
H4K20me1	10	5.933	0.000^***†^	0.017
**Promoters**				
prom 300 all	10	4.172	0.002**	0.009
prom 300 notata	10	3.309	0.009**	0.005
prom 300 tata	10	1.413	0.191	0.023
prom core all	10	5.813	0.000^***†^	0.021
prom core notata	10	1.172	0.271	0.003
prom core tata	10	13.214	0.000^***†^	0.125
promoter all	10	2.576	0.030*	0.015
promoter no tata	10	1.361	0.207	0.005
promoter tata	10	3.830	0.004**	0.055
**Regulatory**				
Human ensembl regulatory	10	−5.806	0.000^***†^	−0.214
Human nontata promoters	10	4.347	0.002**	0.014
Human ocr ensembl	10	57.650	0.000^***†^	0.085
**Splice sites**				
Reconstructed	10	6.034	0.000^***†^	0.051
Splice sites acceptors	10	35.000	0.000^***†^	0.306
Splice sites all	10	38.313	0.000^***†^	0.227
Splice sites donors	10	13.677	0.000^***†^	0.311
**Taxonomic**				
Human or worm	10	3.848	0.004**	0.008
**Transcription factors**				
mouse tfp 0	10	−10.253	0.000^***†^	−0.037
mouse tfp 1	10	−3.525	0.006**	−0.006
mouse tfp 2	10	−10.762	0.000^***†^	−0.084
mouse tfp 3	10	−5.847	0.000^***†^	−0.083
mouse tfp 4	10	0.899	0.392	0.006
human tfp 0	10	4.766	0.001^**†^	0.024
human tfp 1	10	1.251	0.243	0.008
human tfp 2	10	5.016	0.001^***†^	0.042
human tfp 3	10	1.101	0.300	0.010
human tfp 4	10	1.363	0.206	0.009
**Virus variant detection**				
virus covid	10	−7.168	0.000^***†^	−0.073

* *P* <0.05.

** *P* <0.01.

*** *P* <0.001.

† Bonferroni corrected (α=0.05/44=0.0011).

**Table 4.** Paired comparison of character-level versus byte pair encoding tokenization on MCC scores across different genomic features in a 4-layer Mamba-DNA model (matched by seed).

Category	t-statistic	*P*-value	Mean difference (char—bpe)
Coding	−0.6642	0.5232	−0.0292
Enhancers	1.2654	0.2117	0.0067
Epigenetic marks	3.6020	0.0005^***†^	0.0092
Promoters	6.0862	0.0000^***†^	0.0289
Regulatory	−1.4478	0.1584	−0.0384
Splice sites	12.4398	0.0000^***†^	0.2235
Taxonomic	3.8482	0.0039^**†^	0.0081
Transcription factors	−2.3517	0.0207*	−0.0111
Virus variant detection	−7.1678	0.0001^***†^	−0.0726

* *P* <0.05 .

** *P* <0.01 .

*** *P* <0.001 .

† Bonferroni corrected (α=0.05/9=0.0056).

### 3.2 Limited overlap between learned BPE tokens and known regulatory motifs

In order to determine if the vocabulary learned by the byte-pair encoding process contains known regulatory motifs, we did a comparison with exact string matching between the BPE tokenizer vocabulary and the JASPAR 2024 CORE transcription factor binding motif database (Rauluseviciute *et al.* 2024) and found that only 1.54% of the learned tokens correspond to annotated regulatory motifs.

## 4 Discussion

While tokenization approaches have been extensively investigated and benchmarked in natural language models, their impacts on gLMs have remained largely unexplored. The distinct characteristics of genomic language relative to natural language necessitate a careful evaluation of the advantages and limitations of various tokenization strategies when applied to genomic data. Our comparison of tokenization approaches on a range of biological tasks provide insights into the importance of modeling choices in machine learning and can guide future model development in the biological sciences.

Our direct comparison of tokenization methods using the Mamba architecture indicates that single-nucleotide resolution is superior for specific downstream tasks, notably promoter detection and splice site prediction. This aligns with research in the NLP community that demonstrates that character-level resolution improves performance on downstream tasks such as spelling and arithmetic tasks (Shi *et al.* 2022, Xue *et al.* 2022, Chai *et al.* 2024, Singh and Strouse 2024). In the case of splice site prediction, canonical splice sites have highly conserved dinucleotide sequences (e.g. GT/AG) at exon–intron boundaries, and a single nucleotide mutation in this region can disrupt splicing function (Wang *et al.* 2019). Similarly, promoters often contain specific nucleotide motifs which can be highly conserved within and between species (Yang *et al.* 2007) and have conserved spatial arrangements in the nucleotide sequences (Kanhere and Bansal 2005). The mean MCC differences that we see between the tokenization methods for promoter detection (Δ=+0.0289) and particularly for splice-site detection (Δ=+0.2235) suggest that at this relatively small parameter size, without the single-nucleotide precision of character tokenization, the model’s ability to predict these features is reduced.

The attention-based models that used subword tokenization perform better on the splice site and promoter detection tasks than the Mamba-BPE model, which may indicate that the attention mechanism better compensates for subword tokenization’s reduced nucleotide-level precision when identifying specific sequence motifs.

The original DNABERT model scored 0.931 on the splice site detection task, very close to the highest performing model, Nucleotide Transformer (0.941), which has significantly more parameters and is trained on a more diverse dataset. Although the overlapping k-mer tokenization used by DNABERT is not character-level tokenization, the single-nucleotide shift between adjacent tokens preserves nucleotide-level information content, making it effectively equivalent to character-level tokenization, and allowing the model to learn at a nucleotide-level resolution. The DNABERT-2 model, which uses BPE tokenization, drops in performance on the same category by 0.096, suggesting that nucleotide-level resolution improves performance in splice site prediction. We postulate that the larger parameter size of the Nucleotide Transformer enables the model to learn more splice site patterns, and its consistent token size (6 nt) may also facilitate the model’s learning of specific distances.

In the Mamba model direct comparison, byte-pair encoding outperforms character tokenization on the SARS-CoV-2 variant classification task. This task challenges the model to differentiate between nine different variant classes, while all other classification tasks have only two or three classes. This could indicate that there are other categories of tasks not tested in this study where BPE may provide significant advantage.

The regulatory and transcription factor tasks had mixed results, with some datasets favoring BPE and some datasets favoring character tokenization, but there was no statistically significant difference at the category level.

In all remaining task categories, we did not observe any meaningful differences in performance. We hypothesize that these tasks likely rely on broader trends in sequence composition. Sequences from different organisms, for example, are not primarily differentiated by specific nucleotide motifs. Instead, other factors like GC content and oligonucleotide frequency distributions have been found to be significantly different between organisms (Karlin and Mrázek 1997). Similarly, prediction of the sites of epigenetic modification of histones has been associated with broader trends in sequence composition rather than the presence of specific motifs (Pham *et al.* 2007). The comparable performance of different tokenizers on these tasks suggests that when predictions depend on broad compositional patterns rather than specific motifs, multiple tokenization approaches can effectively capture the relevant features.

Our motif analysis indicated that the byte-pair encoding algorithm, as it is currently being applied to genomic sequences, does not efficiently learn a vocabulary of known genomic motifs. One probable explanation is that although motifs are important, they are not frequent, and the BPE algorithm builds the vocabulary based on the most frequent words that appear in the training dataset. The training sets used for the published gLMs are primarily made up of randomly selected DNA segments. Curating these training sets to contain a higher proportion of known regulatory motifs or regions may improve the model’s ability to learn these motifs.

### 4.1 Limitations and future work

To limit the scope of this study, we did not investigate different vocabulary sizes for the subword tokenizers, and did not explore every available subword tokenizer. We focused on the tokenizers currently used in published pretrained models and fixed the vocabulary size at 4096 based on the recommendation of Zhou *et al.* (2024). We tested only classification tasks and no regression or generation tasks were compared. The short context window in current attention-based gLMs precluded us from a direct comparison between character-based and BPE, however, with new model architectures expanding this context window, a direct comparison of these tokenization methods in an attention-based model should be completed. In addition, compared to large language models, the model sizes of all published gLMs are relatively small, and they have been trained primarily on eukaryotic species. More study is needed to evaluate how tokenization decisions will affect models with significantly more parameters and models trained on data from other taxonomic domains. The focus of this study was tokenization, but our results illustrate significant performance differences between model architecture decisions, including differences between the different state space model architectures. These differences should be explored more fully in future work.

## 5 Conclusions

In conclusion, our experiments demonstrate that the selection of tokenization methods substantially influences model performance on downstream genomic tasks. The performance of the BPE tokenizer on the difficult nine-category discriminatory task of SARS-CoV-2 variant classification illustrates that on more challenging genomic tasks, BPE tokenizers may have an advantage beyond compression. We acknowledge that the limited performance of BPE in our study could also be attributed to the limited parameter size of current gLMs. Although BPE has proven valuable in natural language processing, our results suggest that it may not be optimal for all genomic classification tasks, particularly those that require the precise identification of biological motifs. This work underscores the critical role of domain-specific knowledge in model development and highlights the necessity for further investigation into gLM tokenization, challenging assumptions carried over from natural language processing. Ultimately, these findings emphasize the potential to develop novel tokenization strategies tailored to the unique characteristics of genomic sequences, potentially incorporating biological priors or adaptive schemes that preserve biologically relevant units, to achieve improved performance.

## Supplementary Material

btaf456_Supplementary_Data

## Data Availability

Detailed results of all benchmarking experiments are available in https://github.com/leannmlindsey/DNAtokenization. Training datasets and pretrained models are available at https://huggingface.co/datasets/leannmlindsey. Datasets and processing scripts are available at doi: 10.5281/zenodo.16287401 and doi: 10.5281/zenodo.16287130.
